# Neural activity modulations and motor recovery following brain-exoskeleton interface mediated stroke rehabilitation

**DOI:** 10.1016/j.nicl.2020.102502

**Published:** 2020-11-19

**Authors:** Nikunj A. Bhagat, Nuray Yozbatiran, Jennifer L. Sullivan, Ruta Paranjape, Colin Losey, Zachary Hernandez, Zafer Keser, Robert Grossman, Gerard E. Francisco, Marcia K. O'Malley, Jose L. Contreras-Vidal

**Affiliations:** a Non-Invasive Brain Machine Interface Systems Laboratory, University of Houston, Houston, TX 77004, USA; b Department of Physical Medicine and Rehabilitation, McGovern Medical School, NeuroRecovery Research Center at TIRR Memorial Hermann, University of Texas Health Science Center at Houston, TX 77030, USA; c Mechatronics and Haptic Interfaces Laboratory, Rice University, Houston, TX 77005, USA; d Houston Methodist Research Institute, Houston, TX 77030, USA; e NSF IUCRC BRAIN, University of Houston, Houston, TX 77004, USA

**Keywords:** ARAT, Action Research Arm Test, BCI, Brain-Computer Interface, BMI, Brain-Machine Interface, CIMT, Constraint Induced Movement Therapy, CONSORT, Consolidated Standards of Reporting Trials, FMA-UE, Fugl-Meyer Assessment for Upper Extremity, JTHFT, Jebsen-Taylor Hand Function test, MCID, Minimal Clinically Important Difference, MRCP, Movement Related Cortical Potentials, ROC, Receiver Operating Characteristics, Brain-machine interface, Stroke rehabilitation, Exoskeletons, Clinical trial, Movement related cortical potentials

## Abstract

Brain-machine interfaces (BMI) based on scalp EEG have the potential to promote cortical plasticity following stroke, which has been shown to improve motor recovery outcomes. However, the efficacy of BMI enabled robotic training for upper-limb recovery is seldom quantified using clinical, EEG-based, and kinematics-based metrics. Further, a movement related neural correlate that can predict the extent of motor recovery still remains elusive, which impedes the clinical translation of BMI-based stroke rehabilitation.

To address above knowledge gaps, 10 chronic stroke individuals with stable baseline clinical scores were recruited to participate in 12 therapy sessions involving a BMI enabled powered exoskeleton for elbow training. On average, 132 ± 22 repetitions were performed per participant, per session. BMI accuracy across all sessions and subjects was 79 ± 18% with a false positives rate of 23 ± 20%.

Post-training clinical assessments found that FMA for upper extremity and ARAT scores significantly improved over baseline by 3.92 ± 3.73 and 5.35 ± 4.62 points, respectively. Also, 80% participants (7 with moderate-mild impairment, 1 with severe impairment) achieved minimal clinically important difference (MCID: FMA-UE >5.2 or ARAT >5.7) during the course of the study. Kinematic measures indicate that, on average, participants’ movements became faster and smoother. Moreover, modulations in movement related cortical potentials, an EEG-based neural correlate measured contralateral to the impaired arm, were significantly correlated with ARAT scores (ρ = 0.72, *p* < 0.05) and marginally correlated with FMA-UE (ρ = 0.63, *p* = 0.051). This suggests higher activation of ipsi-lesional hemisphere post-intervention or inhibition of competing contra-lesional hemisphere, which may be evidence of neuroplasticity and cortical reorganization following BMI mediated rehabilitation therapy.

## Highlights

• Motor intention based arm training targets activity-dependent neuroplasticity.
• 80% of stroke participants recovered clinically relevant functional movements.
• Ipsi-lesional, delta-band EEG activity was highly correlated with motor recovery.
• Results suggest higher activation of ipsi-lesional hemisphere post-intervention.

## Graphical abstract

**Figure:**
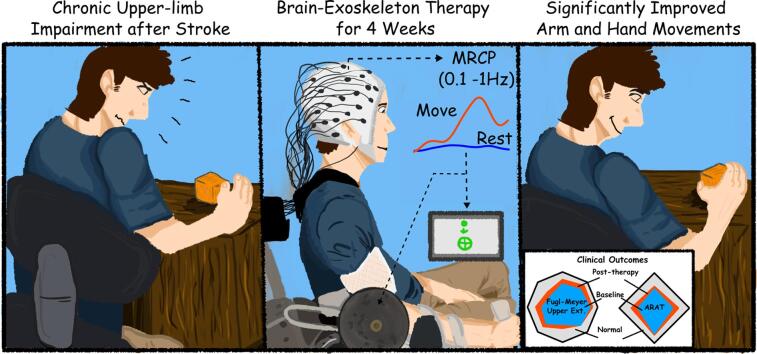

## 1 Introduction

Upper-limb motor weakness occurs in 77% of first time and 55 – 75% chronic stroke survivors and significantly affects their quality of life (Coscia et al., 2019, Lawrence et al., 2001). Regaining arm and hand function is an essential part of achieving independence in daily life and therefore is a major goal of rehabilitation programs. While most traditional rehabilitative strategies are using bottom-up approaches by incorporating training of distal body parts to influence neural systems (Belda-Lois et al., 2011), e.g., constraint induced movement therapy (CIMT) (Wolf et al., 2008), robotic arm training (Lo et al., 2010), bilateral arm training (Whitall et al., 2000), or functional electrical stimulation (Makowski et al., 2014), a number of studies have addressed clinical effects of top-down approaches, e.g., brain stimulation (Dimyan and Cohen, 2010, Liew et al., 2014), motor imagery (López et al., 2019) and brain-computer interface (BCI) (Daly and Wolpaw, 2008) to induce neuroplastic changes in the sensorimotor network, especially in stroke survivors with severe motor deficits.

Brain-machine/computer interface (BMI/BCI) can improve treatment benefits when combined with robotic and muscular stimulation based neurorehabilitation therapies, by capitalizing on the principles of Hebbian plasticity (Soekadar et al., 2015). Indeed, previous clinical studies that combined motor imagery based BMIs with upper-limb arm and hand exoskeletons or electrical muscle stimulation achieved significantly better motor improvement compared to sham or control groups (Ang et al., 2014, Biasiucci et al., 2018, Frolov et al., 2017, Pichiorri et al., 2015, Ramos-murguialday et al., 2013). Despite these promising findings, evidence of cortical changes following neurorehabilitation therapy remain largely unproven, and a neural correlate (or biomarker) that can predict the extent of motor recovery still remains elusive (Stinear, 2017). To address this deficit, Ramos-murguialday *et al.* (Ramos-murguialday et al., 2013) used functional MRI and found post-therapy activations in the ipsi-lesional motor and pre-motor cortices to be correlated (ρ = 0.55) with Fugl-Meyer Assessment for Upper Extremity (FMA-UE) scale. Ang *et al.* (Ang et al., 2014) found the revised Brain Symmetry Index to be inversely correlated to motor improvement, suggesting that bilateral activations of cortical hemispheres led to better recovery (ρ = -0.62). Others have reported increased resting state functional connectivity and integrity of white matter tracts (via diffusion tensor imaging) within the motor areas of both hemispheres following BMI mediated stroke rehabilitation (Biasiucci et al., 2018, Rathee et al., 2019, Song et al., 2015).

In this study, we explored the relationship between movement related cortical potentials (MRCP) and motor recovery, following 12 sessions of BMI-enabled robot-assisted stroke rehabilitation. It was hypothesized that MRCP amplitude and latency (i.e., duration of MRCP prior to movement onset) would increase, on account of increased activation of the ipsi-lesional hemisphere or inhibition of competing contra-lesional hemisphere, following motor relearning and cortical reorganization (Yilmaz et al., 2015). Further, to increase patient engagement and strengthen MRCPs, the BMI algorithms were optimized to detect MRCPs in single-trials using our previously published method (Bhagat et al., 2016). Preliminary findings of our clinical trial, reporting the improvements in movement quality and arm function from initial 6 participants, were published previously (Sullivan et al., 2017). In this paper, we present a comprehensive analysis from 10 participants by determining longitudinal efficacy of EEG-based BMIs, as well as by evaluating changes in brain activity, motor recovery, and movement quality following BMI-exoskeleton therapy.

## 2 Methods

A single-arm clinical study (ClinicalTrials.gov #NCT01948739) was conducted to evaluate the efficacy of BMI enabled exoskeletons on stroke recovery and brain activity. The study procedures were approved by the Institutional Review Boards of University of Houston, Rice University, University of Texas Health Science Center at Houston, and the Houston Methodist Hospital at Houston, Texas. All participants provided informed consent in accordance with the Declaration of Helsinki.

### 2.1 Study participants

Between 2013 and 2018, 160 individuals were screened for eligibility based on following inclusion criteria: first time subacute and chronic stroke (i.e. at least 3 months since injury); stable baseline arm function (see below); hemiparesis of upper extremity (manual muscle testing of at least 2 but no more than 4 out of 5 in elbow and wrist flexors); no joint contracture or severe spasticity; no neglect that would preclude participation in the training protocol; presence of proprioception; no history of neurolytic procedure in the past four months; and no contraindication to MRI. Persons with orthopedic limitation of upper extremity that would affect motor performance; lack of motivation due to untreated depression were excluded from the study. To evaluate baseline arm function stability, FMA-UE assessment was performed at screening and was repeated one month later. A participant was enrolled only if the difference in FMA-UE scores at these visits was ≤3 points (Klamroth-Marganska et al., 2014).

**Fig. 1:**
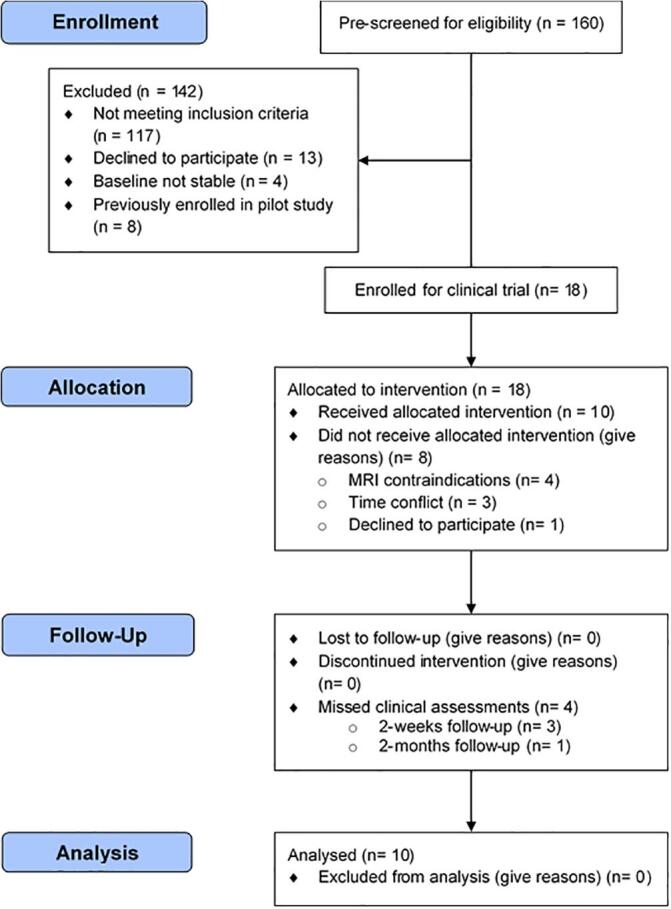
CONSORT flow diagram showcasing patient recruitment, intervention and follow-ups.

Among the participants excluded at screening (n = 142), 117 did not meet the inclusion criteria, 4 did not have a stable baseline, and 13 declined to participate. In addition, 8 individuals that previously participated in our pilot study (Bhagat et al., 2016) for the clinical trial were excluded, since they were familiar with BMI-exoskeleton therapy paradigm. Subsequently, eighteen participants enrolled in the study and were assigned to the BMI-exoskeleton therapy group, and there was no control group. Among these participants, 10 individuals completed the protocol. Participants who dropped out of the study had MRI contraindication (n = 4), could not commit time to participate in all therapy and assessment sessions (n = 3), or were not interested in participating (n = 1). The enrollment and intervention details following the Consolidated Standards of Reporting Trials (CONSORT) flow diagram are shown in Fig. 1.

The study cohort consisted of participants with either cortical (n = 4), subcortical (n = 4), or both cortical and subcortical lesions (n = 2). Specific details regarding the location of stroke lesions, as determined by physicians after reviewing T1-weighted MRI scans, are provided in Supplementary Table S1. And in Table 1 below, we present demographics and baseline characteristics of participants who completed the study. Additionally, the average grip and pinch strengths for our participant pool were 11.13 ± 8.7 kg and 4.48 ± 2.3 kg, respectively. According to (Woytowicz et al., 2017) classification of impairment severity, the participants can be further grouped as severe-moderate impaired (baseline FMA-UE ∈[16, 34]) or moderate-mild impaired (baseline FMA-UE ∈[35, 53]), which is also highlighted in Table 1.

**Table 1:** Demographics and baseline assessments of study participants.

### 2.2 Study protocol and experiment design

**Fig. 2:**
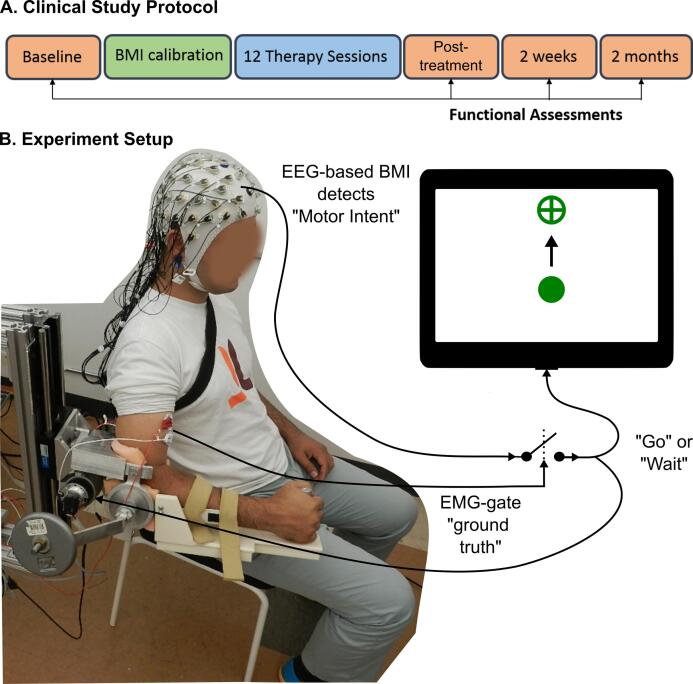
**EEG-based BMI control of MAHI exoskeleton for stroke rehabilitation.** A) Timeline for the clinical study protocol. B) Schematic representation of the experiment setup, showing a stroke participant’s impaired elbow being trained by the MAHI Exo-II, while EEG and EMG activity are recorded. In this BMI scheme, successful detection of motor intent from EEG is validated against residual EMG activity from impaired arm, before a Go or Wait command is issued to the exoskeleton. A computer screen in front of the participant, cues start and end of trial and provides simultaneous visual feedback of the movement.

The clinical trial protocol consisted of 14–15 sessions and 5 functional assessments (Fig. 2A). The initial 2 sessions were used for calibrating the BMI algorithm (see Sec. 2.3) to each participant. Participants P2 and P8 underwent an additional calibration session to fine-tune the BMI classifier’s parameters. Once calibrated, the BMI-exoskeleton therapy was provided for 12 sessions, 3 times per week, for 4 weeks. Participant P10 was unavailable during weekdays and hence, his sessions were conducted on the weekends for 6 weeks. The functional assessments were performed twice at baseline as described earlier and once post-treatment, as well as at 2-weeks and 2-months follow-ups.

The primary outcome measures were functional improvement in arm and hand movements using FMA-UE test, modulation in neural activity as measured by EEG, and improvement in movement quality as determined from the exoskeleton’s kinematics. The secondary outcomes assessed motor recovery using additional clinical scales such as Action Research Arm test (ARAT), Jebsen-Taylor Hand Function test (JTHFT), pinch and grip strengths. FMA-UE score is comprised of 8 scoring items, namely arm movements involving flexor synergy, extensor synergy, combined synergies (e.g. move hand to lumbar spine), out of synergy (e.g. shoulder abduction to 90°, while elbow is at 0° and forearm is pronated), hand, wrist, speed/co-ordination, and reflexes (K. J. Sullivan et al., 2011). Likewise, ARAT scores are the aggregate of 4 subscales: grasp, grip, pinch, and gross movements (Yozbatiran et al., 2008). Additionally, we recorded surface electromyography (EMG) from biceps and triceps muscles of both impaired and unimpaired arms to determine if participants exhibited global synkinesis or motor irradiation (Hwang et al., 2005), but also to provide a ‘ground truth’ for the BMI output (Fig. 2B).

Each therapy session lasted 3 to 3.5 h and included EEG preparation (~45 min.), daily kinematic assessment (~15 min.), therapy time (~2 h), and breaks as needed. During therapy, participants were presented with a center-out reaching task on a computer screen to train their elbow flexion and extension movements, while their impaired arm was supported by the MAHI Exo-II exoskeleton (Fitle et al., 2015). To perform the movement, the participants were instructed to “first think about the movement and then gently attempt to move their arm”. Each trial lasted up to 15 s, and the participants could attempt to move multiple times in a trial. If the BMI algorithm successfully detected the motor intention, which was corroborated by EMG activity in the prime muscles, then the exoskeleton was triggered to assist in the movement; otherwise the exoskeleton remained stationary and resisted the movement. This protocol enforced the participants to remain mentally engaged in the task in order to maximize the benefits of the BMI-exoskeleton therapy. A target appeared on the screen either in the upward or downward position at random, corresponding to elbow flexion and extension movements, respectively. Once the target was hit, the exoskeleton automatically returned to center, and after a randomized resting interval (4–6 s), the next trial was presented. Typically, participants practiced 60–180 trials per session (median = 160, IQR = 20), and the number of repetitions increased once they became proficient in controlling the BMI and their fatigue diminished.

### 2.3 BMI algorithm

Our BMI algorithm was based on methods developed previously, wherein an EEG-based classifier’s predictions were gated with residual EMG activity from the impaired arm, before triggering an exoskeleton’s movement (Bhagat et al., 2016). To detect motor intent we identified movement related cortical potentials from delta-band EEG rhythms (0.1 – 1 Hz), using a Go vs. No-go Support Vector Machine (SVM) classifier (Lotte et al., 2007). The classifier was trained for each participant using pre-recorded calibration data, in which they voluntarily moved or triggered movement of the exoskeleton with their impaired arm, while performing motor imagery.

Unlike the previous study, wherein we handpicked the EEG channels that were fed to the classifier, here we automated the channel selection process. First, we visually short-listed EEG channels that contained MRCPs from grand averaged movement epochs. Next, we used backward elimination and dropped channels that were less relevant for classification, as determined from the mutual information between class labels and feature vectors (Lan et al., 2005, Peng et al., 2005). The automatic channel selection was introduced in order to select only a subset of MRCP channels that contributed to class discriminability and thereby, had an impact on the classifier’s performance. Grand-averaged MRCPs measured for each participant during calibration and outcomes from the automatic channel selection process are presented in supplementary materials (Figures S3–S12).

The training algorithm also automatically selected the optimal feature extraction window length using receiver operating characteristics (ROC) curves (Fawcett, 2006). This was achieved by training the classifier offline for different window lengths ranging from 100 ms to 1 s, in 100 ms increments. In each iteration, an ROC curve was obtained using confusion matrices and eventually, the window length corresponding to the classifier with maximum area under ROC curve was considered optimal for that participant.

The online BMI performance was further improved by tuning 2 parameters: the classifier’s prediction probability estimate ($\tau_{c}$) and number of consecutive Go predictions required before intent is asserted ($N_{c}$) (Bhagat et al., 2016). Parameters $\tau_{c}$ and $N_{c}$were initially set at 0.5 and 3 respectively, and increased up to 1 and 10 until the participants achieved high accuracy. Once tuned, the BMI classifier and its parameters were fixed for 12 therapy sessions. For configuring the EMG-gate, a simple threshold detection technique was employed. Under this technique, RMS values for EMG signals from impaired hand were baseline corrected by subtracting the mean value over a 30 s resting period. The resulting signals were then compared against an empirically determined threshold, typically 5 – 30 units above baseline. The EMG thresholds however, did require to be readjusted between sessions and sometimes within a session, to overcome offsets from poor contact with the skin or from brushing against the exoskeleton’s braces.

The online BMI algorithm was implemented such that EEG was the primary deciding factor in the classification of motor intent. Once a positive prediction was made by EEG classifier, then the algorithm checked to see if EMG activity in either flexion or extension muscles is greater than threshold within the next 1 sec and only then the exoskeleton was triggered to assist in the movement.

### 2.4 Computation of post-treatment MRCP changes

To quantify changes in neural activity as a result of therapy, we looked at differences in grand averaged MRCPs between the initial and final closed-loop BMI therapy sessions. MRCPs were calculated with respect to movement onset times identified from EMG activity of the impaired hand. For this, EMG signals were denoised using Teager-Kaiser energy operator, low-pass filtered (0.5 Hz, 4th order Butterworth), standardized, and then compared against a threshold of 0.5 standard deviation to identify intervals of either flexor or extensor contraction (Tenan et al., 2017). Contraction intervals larger than 1 s were retained for further analysis and their time of onset was utilized to segment EEG epochs for calculating MRCPs. This approach ensured that the MRCPs were measured with respect to true movement onset and independent of the classifier’s predictions. To obtain a sufficient number of trials for averaging, we combined EEG epochs from the first 2 and final 2 therapy sessions and then computed their difference. Further, we looked at difference in MRCP peak amplitudes and latency from scalp EEG electrodes located over the motor cortex, specifically, central (C_z_, C_1_- C_4_), fronto-central (FC_z_, FC_1_ – FC_4_) and centro-parietal electrodes (CP_z_, CP_1_ – CP_4_). Further, to account for left hand vs. right hand impairment, the electrode locations were flipped for individuals with right hand impairment. Finally, MRCP latency was defined as time difference starting from 50% of peak amplitude until the time of movement onset (see Supplementary Fig. S1) (Muller-Gethmann et al., 2000).

### 2.5 Data and statistical analysis

The benefit of BMI-enabled exoskeleton therapy was assessed with two objectives, namely improvement in patient engagement (measured as a participant’s ability to reliably operate a BMI) and improvement in motor function (measured via changes in neural activity, clinical scores, and movement kinematics). BMI performance was quantified per session in terms of prediction accuracy, false positives, early detection time, and user feedback. Prediction accuracy was determined based on the fraction of successful trials from total trials, while to calculate false positives, we used catch trials that asked participants to intentionally remain idle during those trials. Our early detection time metric measured how far in advance the BMI could predict movement from EEG alone, before a participant physically tried to move their impaired arm (as seen from EMG activity). The participants’ approval rating of the BMI’s decisions was assessed using a 3-point Likert Scale, with a scoring scale of 3 = Approve, 2 = Not sure, and 1 = Disapprove. To compare offline vs. online BMI performance metrics we used Wilcoxon rank sum test, since the data was non-normal and had unequal sample sizes.

To test for statistical significance of motor recovery based on clinical assessments, one-way mixed effects analysis with repeated measures was used. The assessment intervals were taken as fixed effect with four levels (Baseline, post-treatment, 2-weeks, and 2-months follow-up). Whereas a between-subject intercept was considered as the random effect. Mixed effects models were selected over conventional repeated measures ANOVA, to compensate for the missing follow-up sessions (Wainwright, 2007). Additionally, an in-depth analysis of FMA-UE and ARAT subscales was conducted to assess which of their scoring items improved amongst participants and how long were the improvements retained post-intervention.

Movement quality improvements were evaluated by comparing kinematic data from initial 2 with final 2 therapy sessions. These metrics included Average Speed, Spectral Arc Length (a frequency-domain measure that increases in value as movements become less jerky (Balasubramanian et al., 2015)), and two metrics related to the shape of the velocity profile: Number of Peaks (a higher number of peaks implies jerkier movement), and Time to 1st Peak (which is usually less than the ideal value of 0.5, or 50% of the total movement duration, when a movement has more than one peak). Due to the non-normality of the data, Wilcoxon signed rank tests were used on the paired differences for each movement quality metric.

All data analysis were performed in MATLAB R2018b, with the exception of mixed effects analysis which was carried out in R (R Core Team, 2017) and its ‘lme4’ package (Bates et al., 2015). The statistical significance criteria was set at *p*-values less than 0.05.

## 3 Results

### 3.1 BMI performance across participants

**Fig. 3:**
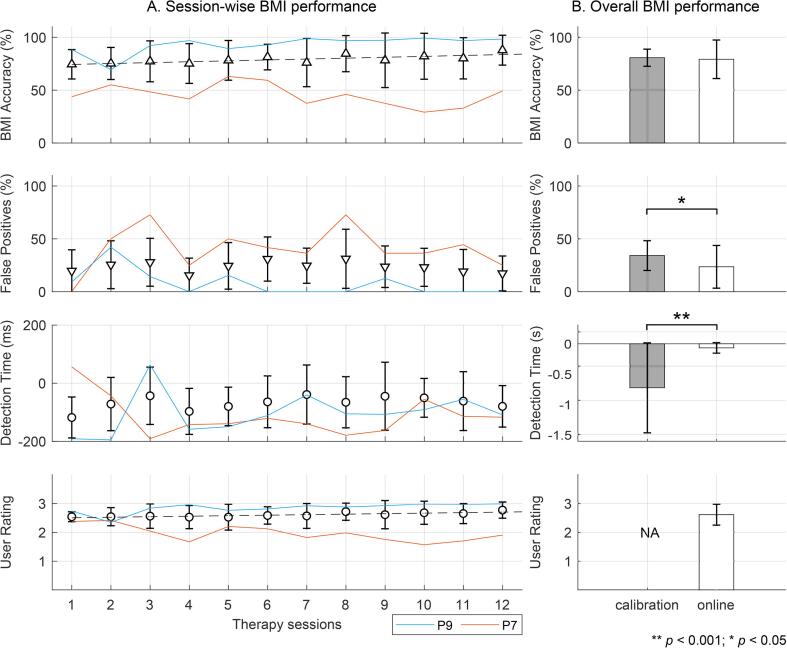
**Longitudinal BMI performance.** BMI performance in 10 chronic stroke survivors over 12 therapy sessions, averaged by session in sub-plot A and averaged by online testing vs. calibration in sub-plot B. From top to bottom, mean ± s.d. values for BMI’s prediction accuracy, false positives, early detection time, and user approval rating are shown. Results from 2 participants (P9 and P7) with best and worst BMI accuracy are overlaid on the plots. Dotted lines indicate statistically significant trends in accuracy and user rating.

During the 4–6 weeks long therapy regime, on average, participants completed 132 ± 22 repetitions per session by triggering the exoskeleton’s movement via the BMI. As seen in Fig. 3A top plot, the average prediction accuracy was consistently better than random chance (=50%) across sessions. During the last 5 therapy sessions, 4 participants achieved greater than 90% accuracy. Overlaid on the plots are BMI performance traces for participants with best (P9) and worst (P7) accuracies across sessions. The remaining plots in this figure demonstrate the BMI’s performance in terms of its ability to avoid false positives, its early detection time, and users’ approval rating. The dotted lines are best fit lines for which the slope was non-zero and statistically significant (*p* < 0.05).

Fig. 3B compares the BMI’s online performance with its offline performance at calibration. Overall the BMI’s accuracy was similar during online and offline testing (79 ± 18% vs. 81 ± 8%, n.s.). The average false positives in the online scenario were significantly smaller than offline (23 ± 20% vs. 34 ± 14%, *p* < 0.05). In offline testing, motor intent could be detected as early as 723 ± 740 ms before onset of movement, while in the online case the early detection of intent could be made only 66 ± 86 ms in advance (p < 0.001). Finally, the average approval rating was high and consistent across users at 2.6 ± 0.4 points on a 3-point Likert scale.

### 3.2 Clinical outcomes

**Fig. 4:**
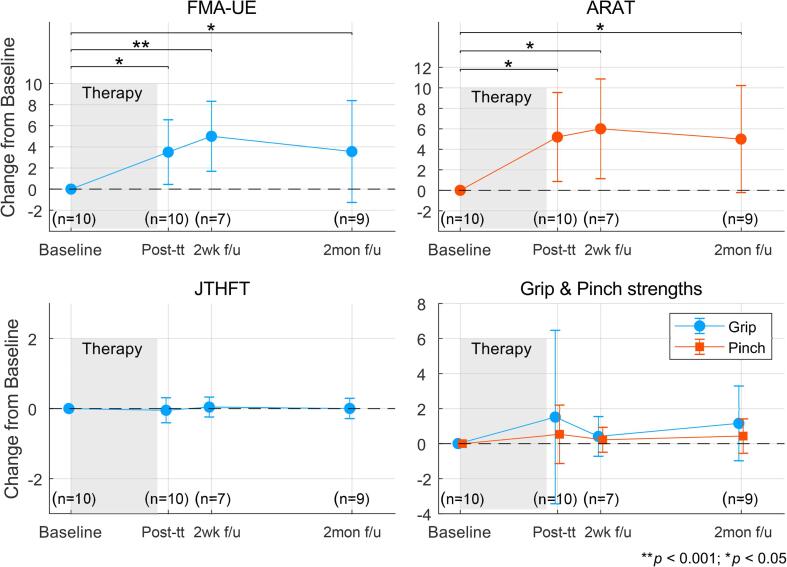
**Motor recovery post-intervention.** Clinical outcome metrics assessed post-treatment (post-tt) and at 2-week (2wk f/u) and 2-months (2mon f/u) follow-ups relative to baseline. Shaded regions indicate the 4 – 6 weeks long intervention period. Underneath each data point, the number of scores that were averaged to calculate the mean value are shown.

Fig. 4 shows changes in clinical metrics from baseline evaluated at different time points: post-treatment, 2-weeks, and 2-months follow-ups. The average change in FMA-UE and ARAT during the entire course of the study were 3.92 ± 3.73 and 5.35 ± 4.62, respectively. Repeated measures mixed effects model analysis confirmed that there were significant improvements from baseline in FMA-UE (F (23.03, 3) = 5.54, *p* < 0.01) and ARAT (F (23.018, 3) = 6.25, *p* < 0.01). Post-hoc analysis revealed that FMA-UE and ARAT scores after treatment and at follow-ups where significantly better than at baseline. Moreover, as shown in Table S2 (supplementary materials), overall 8 participants achieved minimal clinically important difference (MCID) after therapy or at follow-ups, based on their FMA-UE and ARAT scores. MCID thresholds for FMA-UE was set as 5.2 points and for ARAT as 5.7 points change from baseline (Lee et al., 2001, Page et al., 2007). No change in JTHFT scores was observed. Marginal improvements in grip and pinch strengths were noted, but these did not reach statistical significance.

### 3.3 Changes in FMA-UE and ARAT scores by subscales

**Fig. 5:**
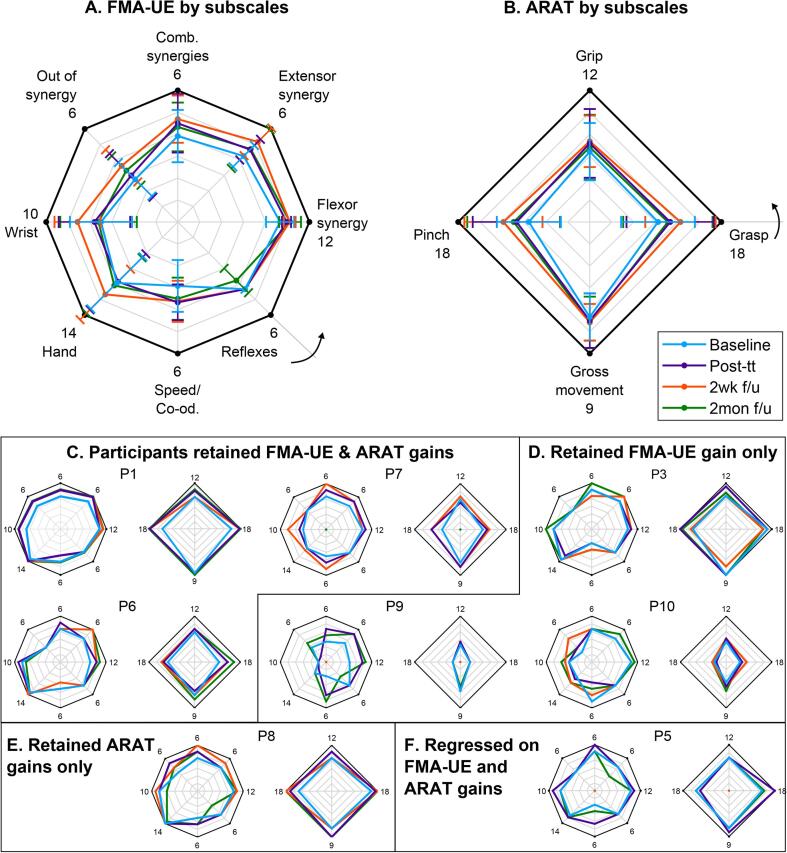
**Detailed breakdown of motor recovery.** Breakdown of FMA-UE and ARAT scores by subscales, shown by averaging across participants (subplot A & B) and individually (subplots C-F) for participants that achieved minimal clinically important difference. Subplots C-F, further group participants based on their FMA-UE and ARAT outcomes at 2 months follow-up. The arrows in subplots A & B indicate the order of administering the test, starting at the first item and then progressing counter-clockwise.

In Fig. 5, we breakdown the FMA-UE and ARAT scores into its constituent subscales. For each of the spider charts shown in the figure, the black outer polygon represents maximum score achievable under each subscale. The maximum score in each scoring item is also stated next to each vertex in subplots A & B, as well as in all remaining subplots. The colored polygons represent the 4 different assessment time points, namely baseline, immediately after treatment, 2-weeks and 2-months follow-ups.

Fig. 5A & B show the mean ± s.d. scores for FMA-UE and ARAT subscales. On average participants improved in movements involving arm synergies, speed, co-ordination, wrist and hand components of FMA-UE, as well as grasp and pinch components of ARAT. The improvements were greatest at 2-weeks assessment, but later regressed and at 2-months follow-up the scores were similar to that of post-treatment, albeit better than baseline. Subplots C-F in Fig. 5 track progress of individual participants that were able to achieve MCID during any of the follow-up assessments. For participants that did not attend a follow-up visit (i.e. P5, P7, and P9), their score was assigned zero in the plots and their most recent assessment score were used for further groupings. Specifically, subplot C groups individuals that retained gains in both FMA-UE and ARAT scores at 2-months follow-up (with the exception of P7). Fig. 5D groups individuals that retained gains in FMA-UE, but either regressed or did not improve on ARAT scores. Similarly, Fig. 5E shows a participant who retained his ARAT scores, but regressed on FMA-UE. Finally, subplot F shows a participant that regressed on both FMA-UE and ARAT scales at 2-months follow-up. Since participants P2 and P4 did not achieve MCID at any point during the study, their score charts are not shown in Fig. 5.

### 3.4 Behavioral outcomes

#### 3.4.1 Motion kinematics

**Fig. 6:**
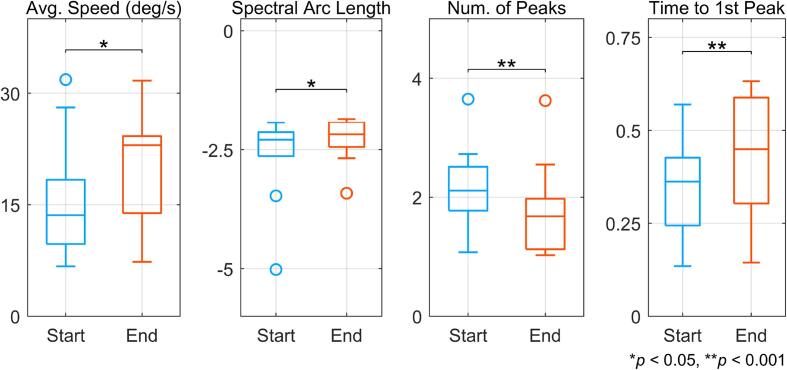
**Improvement in movement quality between start and end of therapy.** Movement quality was derived from joint angle velocity using various kinematic metrics. For all metrics except Number of Peaks, an increase in value corresponds to improvement.

In Fig. 6, boxplots compare movement quality metrics between the start and end of therapy sessions. Using a single-sided Wilcoxon signed rank test, the median values for Average Speed, Spectral Arc Length, and Time to 1st Peak were significantly higher at the end of the therapy. Likewise, the number of peaks were significantly lower at the end of the therapy. Median values for Average Speed increased from 13.6 deg/s to 23 deg/s (*p* < 0.05) and Spectral Arc Length increased from −2.29 to −2.17 (p < 0.05). The median Number of Peaks decreased from 2.11 to 1.68 (*p* < 0.001), which suggests that movements at the end of therapy were less jerky. Also, the median Time to 1st Peak increased from 0.36 to 0.45 (*p* < 0.001), which indicates well-balanced movements (ideal value = 0.5) were achieved post-therapy completion.

#### 3.4.2 Presence of global synkinesis

Bilateral surface EMG analysis revealed that the involuntary co-activation of unimpaired arm when using paretic arm, also known as global synkinesis phenomenon, existed in 2 participants: P4 (baseline FMA-UE = 21, ARAT = 4) and P8 (baseline FMA-UE = 49, ARAT = 42). As seen from the normalized bilateral EMG traces in Supplementary Fig. S2, synkinesis was primarily observed during elbow extension, while it was absent during elbow flexion. Moreover, no change was observed in the extent of co-activation of the unimpaired arm between the start and end of therapy.

#### 3.4.3 Correlation of MRCP amplitude and latency with clinical outcomes

**Fig. 7:**
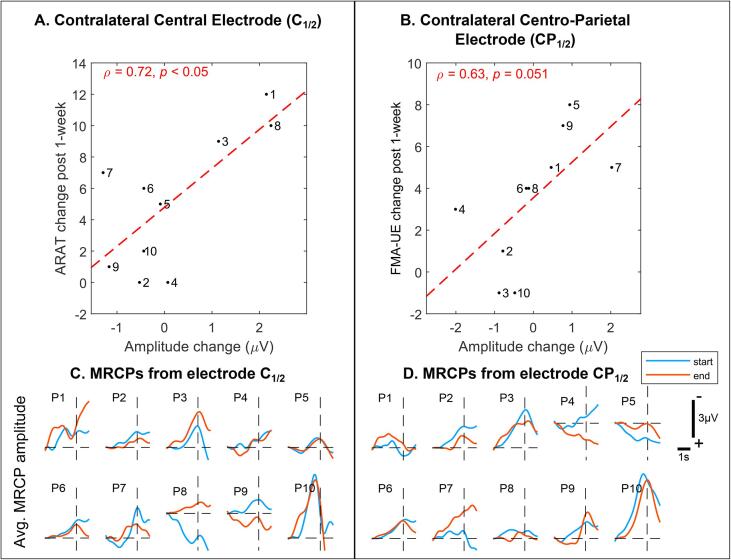
**Correlation (ρ) between MRCP amplitude and functional assessment scales.** Subfigures A & B compare MRCP amplitudes from central and centro-parietal EEG electrodes with clinical outcomes. In these figures, numbers represent participant I.Ds and the dashed lines represent regression lines between changes in MRCP amplitude versus clinical scores. Subfigures C & D show MRCPs recorded from all the participants at start and end of therapy. Note, MRCPs are aligned with respect to movement onset (t = 0 s).

We correlated changes in FMA-UE and ARAT scores post-treatment with differences in MRCP signals, corresponding to initial and final therapy sessions. As seen in Fig. 7 top row, MRCP amplitude from the contralateral EEG electrodes highly correlated with functional assessment scores. Specifically, change in average MRCP amplitude for contralateral central electrode (i.e. C_1_ or C_2_ depending on impaired side and abbreviated as C_1/2_) significantly correlated with ARAT scores (ρ = 0.72, *p* < 0.05). Likewise, correlation between MRCP amplitude from contralateral centro-parietal electrode (i.e. CP_1_ or CP_2_ depending on impaired side and abbreviated as CP_1/2_) and FMA-UE scores, was tending towards significance (ρ = 0.63, *p* = 0.051). No significant correlation between MRCP latencies and clinical outcomes was observed. Fig. 7C & D plot the averaged MRCP signals from the initial and final therapy sessions for all participants, corresponding to central and centro-parietal EEG electrodes.

## 4 Discussion

Cortical reorganization and motor recovery following stroke are contingent on ensuring active user engagement and participation during rehabilitation, to promote activity-dependent neuroplasticity (Venkatakrishnan et al., 2014). Towards this extent, BMI-based neurorehabilitation therapies have performed arguably better at engaging the user and achieving better functional outcomes than any other contemporary rehabilitation therapies (e. g. CIMT, robot-assisted or neuromuscular stimulation alone, etc.) (Cervera et al., 2018). In the same light, this study confirmed that BMI-enabled robot-assisted upper-limb therapy resulted in improved motor function for a majority of the participants with chronic stroke, as determined from post-treatment, 2-weeks, and 2-months assessments.

Specifically, functional metrics that are typically associated with arm/hand movements and co-ordination, i.e. FMA-UE and ARAT, improved as a result of therapy (7 participants with moderate-mild impairment and 1 with severe-impairment showed some level of motor recovery by the end of the intervention). Whereas, metrics associated with hand strengthening and speed, such as JTHFT, grip and pinch strengths remained stable. Since the BMI-enabled MAHI Exo-II exoskeleton was primarily targeting elbow training, this result is expected. However, as seen in Fig. 5A & B, the effects of elbow training generalized to positive improvements in wrist and hand subscales of FMA-UE and pinch and grasp subscales of ARAT outcomes. This is likely due to the enhanced somatosensory feedback provided by neurorehabilitation therapy, as well as increase in a participant’s ability to use their hand and wrist, following recovery of their proximal joints (Ang et al., 2014). It is interesting to note that participants’ arm recovery continued to improve at 2-weeks, while other studies did not see any change during this time window e.g. Klamroth-Marganska, et al, 2014 (Klamroth-Marganska et al., 2014) and Biasiucci et al, 2018 (Biasiucci et al., 2018). These improvements might be related to increased arm use in daily life. Moreover, maintenance of improvement at 2-months (as compared to baseline) is indicative of continued treatment benefits in long-term. No adverse events directly related to the intervention were reported, although one participant (P5) experienced unexplained tiredness, forgetfulness, and excessive decline in motor performance, 2 months after therapy (see Fig. 5F).

While clinical outcomes are indisputable evidence of motor relearning, often these are imperceptive to cortical changes at sub-clinical levels. Hence, to determine the efficacy of any neurorehabilitation therapy, it is important to identify neural correlates or biomarkers that can explain and even predict post-treatment clinical outcomes. Indeed, previous studies have identified neural correlates based on the BOLD response (Ramos-murguialday et al., 2013), white matter tract anisotropy (Song et al., 2015), brain symmetry index (Ang et al., 2014), and sensorimotor rhythms’ spectral power (Bundy et al., 2017). Our analysis of MRCPs from start to end of therapy showed that participants who improved in motor function were characterized by modulation in MRCP amplitudes from the contralateral EEG electrodes that were highly and positively correlated with functional assessment scores. More specifically, MRCP amplitudes from the primary motor cortex and post-central gyrus (Brodmann Areas 4 & 7) contralateral to the impaired arm, correlated with ARAT (ρ = 0.72, *p* < 0.05) and FMA-UE (ρ = 0.63, *p* = 0.051) scores, respectively (Koessler et al., 2009). However, no significant correlation with MRCP latency was observed. Since MRCP amplitude is believed to encode information about computational effort and attention (Cui and MacKinnon, 2009), increase in MRCP amplitude suggests higher activation of the ipsi-lesional hemisphere or inhibition of the competing contra-lesional hemisphere. However, it is still unclear, if higher activation of ipsi-lesional hemisphere is a consequence of cortical reorganization or neuroplasticity, and should be explored in a future study.

Interestingly, even though our participants performed a small number of physical movements per session (132 ± 22), their functional and kinematic outcomes were comparable to high-intensity robot-only therapies (Klamroth-Marganska et al., 2014, Lo et al., 2010). This was likely facilitated by the BMI’s consistent decoding accuracy (avg. = 79 ± 18%), low false positives (23 ± 20%) and early detection latency (−66 ± 86 ms). This in turn allowed the exoskeleton to seamlessly respond to the participant’s volitional movement intent and provide causal afferent sensory feedback, thereby promoting cortical plasticity. It is important to note that while motor intent could be predicted before the physical movement onset, the online detection occurred much later (−66 ± 86 ms) than offline detection (−723 ± 740 ms). This difference in detection latencies arises more from the classifier’s tradeoff between sensitivity/specificity than due to MRCP variability. In the offline scenario, the classifier parameters: prediction probability threshold ($\tau_{c}$) and number of consecutive Go decisions required ($N_{c}$), were fixed at 0.5 and 3, respectively. Whereas, during the online BMI fine-tuning, these parameters where gradually increased to improve the classifier’s specificity. This inadvertently made the classifier take longer to declare a Go decision, which resulted in shorter early detection time.

Our study did have a few shortcomings. The absence of a control group prevented us from understanding the individual benefits of BMI and robotic therapy alone. However, we ensured that the participants enrolled had a stable baseline and any improvements can be attributed to the combined effect of BMI plus robotic therapy. Our sample size was small (n = 10), which prevents us from generalizing the outcomes to a larger sample. This was in part to our narrow inclusion criteria, which excluded about 75% of the participants that were screened. The BMI control was limited to one-dimensional (Go vs. No-go), which might not have been engaging enough for some of the participants (e.g. P7). For future participants, it should be prioritized to achieve multi-dimensional BMI control and combine it with virtual or augmented reality, to provide an immersive learning environment.

Most existing BMIs make use of mu or beta band power modulations (event-related desynchronization), whereas the BMI presented in this study used MRCPs to detect movement intentions, which was further corroborated by EMG activity in the prime muscles. Because these BMI setups occur in different frequency bands (higher vs. lower frequencies, respectively) and differ in the domain they are computed (frequency vs. time-domain, respectively), it is not possible to directly compare them because they make use of entirely different physiological features. Finally, the validity of MRCPs as a neurophysiological marker for subclinical improvement must be taken with caution. Besides the small study cohort, the bottom plots in Fig. 7 depicts high intra-subject variability in amplitudes. Also, there were 3 participants (P5, P6, and P7) whose MRCP-amplitude even decreased post-therapy, while their ARAT-scores increased. Nonetheless, our study found compelling evidence for clinical efficacy of BMI-enabled robot-assisted rehabilitation.

## CRediT authorship contribution statement

**Nikunj A. Bhagat:** Methodology, Software, Investigation, Data curation, Formal analysis, Writing - original draft. **Nuray Yozbatiran:** Methodology, Investigation, Formal analysis, Writing - review & editing. **Jennifer L. Sullivan:** Software, Investigation, Data curation, Formal analysis. **Ruta Paranjape:** Investigation, Data curation. **Colin Losey:** Investigation. **Zachary Hernandez:** Investigation. **Zafer Keser:** Investigation. **Robert Grossman:** Investigation, Writing - review & editing. **Gerard E. Francisco:** Conceptualization, Funding acquisition, Supervision, Investigation. **Marcia K. O'Malley:** Conceptualization, Funding acquisition, Supervision, Writing - review & editing. **Jose L. Contreras-Vidal:** Conceptualization, Funding acquisition, Supervision, Writing - review & editing.

## Competing interests

N.B. and J.C. have a patent issued (US10,092,205 granted October 9, 2018), which presents methods for detecting motor intentions from EEG signals, including MRCPs. All the remaining authors report no competing interests.
