# Supplementary material for: Neural activity modulations and motor recovery following brain-exoskeleton interface mediated stroke rehabilitation
Source: Neuroimage Clin. 2020 Nov 19;28:102502. doi: 10.1016/j.nicl.2020.102502 (PMC7749405; doi:10.1016/j.nicl.2020.102502)
Supplement: Supplementary data 1 [file mmc1.pdf]

Supplementary Materials

Table S1. Stroke Etiology and Lesion Location of Study Participants

Patient I.D.	Stroke Type & Location
P1	Left frontal and parietal lobes hemorrhagic infarcts
P2	Right superior temporal gyrus, inferior and middle frontal gyri, right precentral gyrus ischemic infarcts
P3	Right thalamocapsular and left posterior thalamus and left posterior putamen ischemic infarcts
P4	Right frontal, parietal and occipital lobes ischemic infarcts
P5	Left corona radiata and putamen, left cerebral peduncle ischemic infarcts
P6	Left frontal lobe and left basal ganglia hemorrhagic infarcts
P7	Left insula, putamen and external capsule hemorrhagic infarcts
P8	Right frontal lobe ischemic infarct
P9	Right external capsule ischemic infarct
P10	Left basilar part of pons hemorrhagic infarct

Table S2. No. of participants achieving MCID with respect to baseline

Assessments	Post-treatment	2-weeks	2-months	**Overall**
FMA-UE	2	4	4	**8** (P1, P3, P5, P6, P7, P8, P9, P10)
ARAT	5	5	3	
# Assessed	10	7	9	**10**

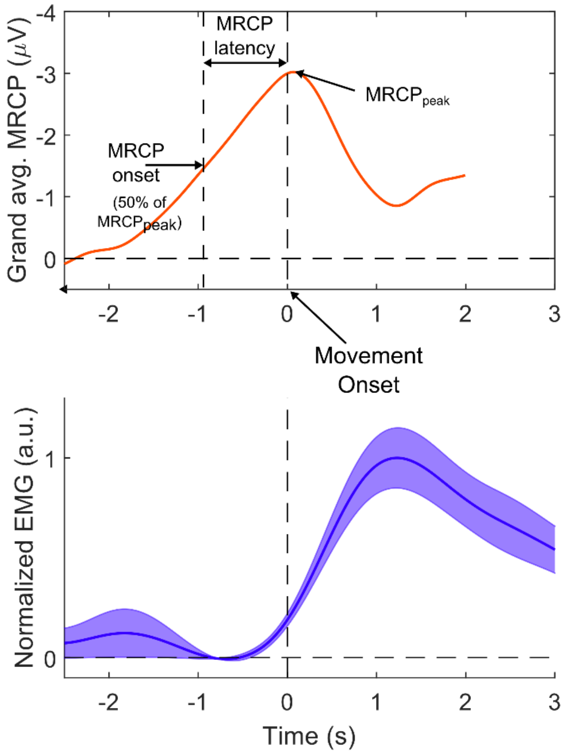

Fig. S1. (Top row) MRCP latency defined as the time difference between MRCP onset and movement onset. MRCP onset is taken as time point corresponding to 50% of grand averaged MRCP peak amplitude. (Bottom row) Normalized EMG activity (mean +/- 95% C.I.) of impaired arm muscles used to identify movement onset (see Methods section for details) shown for an example participant.

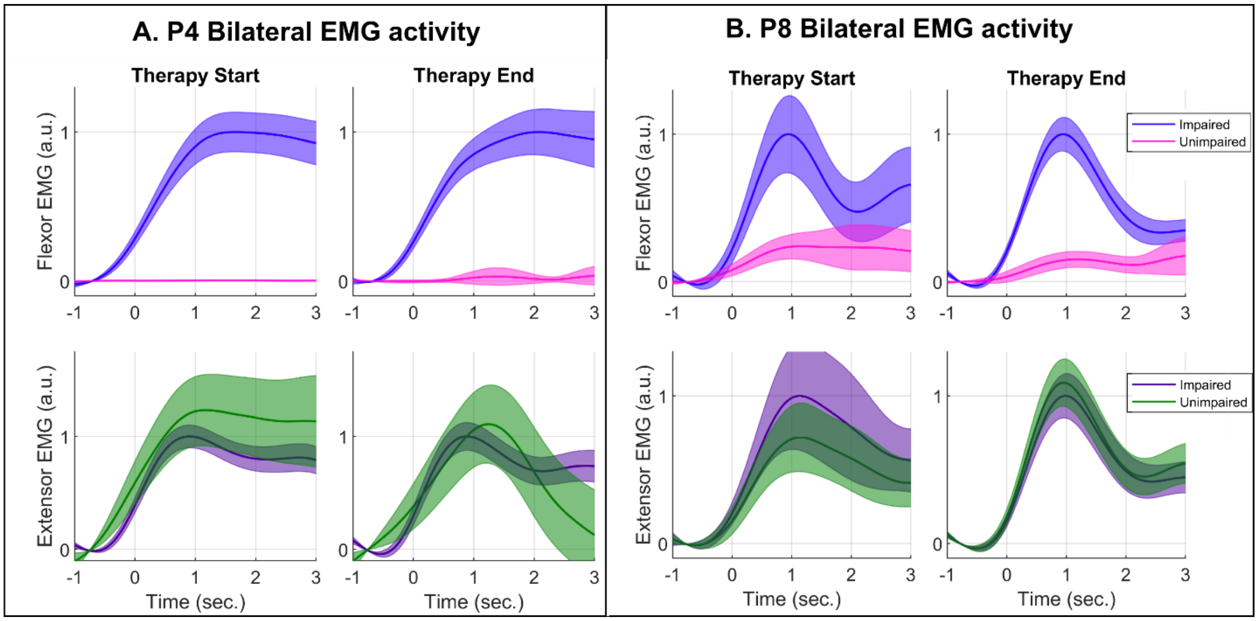

Fig. S2. Normalized EMG traces (mean +/- 95% C.I.) showing presence of global synkinesis in 2 of 10 study participants (P4 and P8) at start and end of therapy. Time (t = 0s) corresponds to voluntary movement onset of the impaired arm.

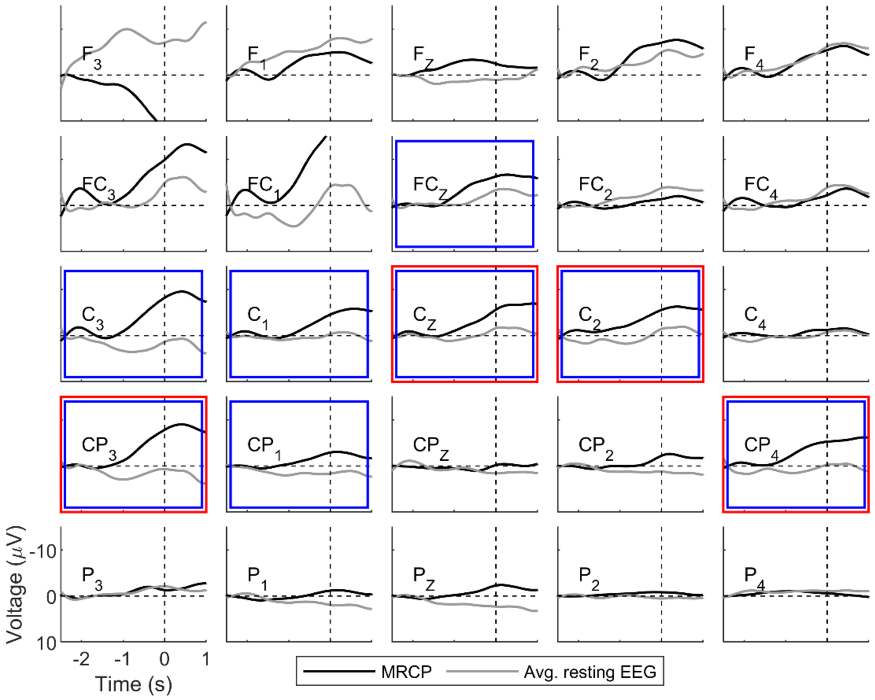

Fig. S3. Grand-averaged MRCPs measured during movement and rest trials for participant P1. Blue boxes indicate visually short-listed MRCP channels. Of these, channels retained by the automatic channel selection algorithm for training the BMI classifier, are encircled by a red box.

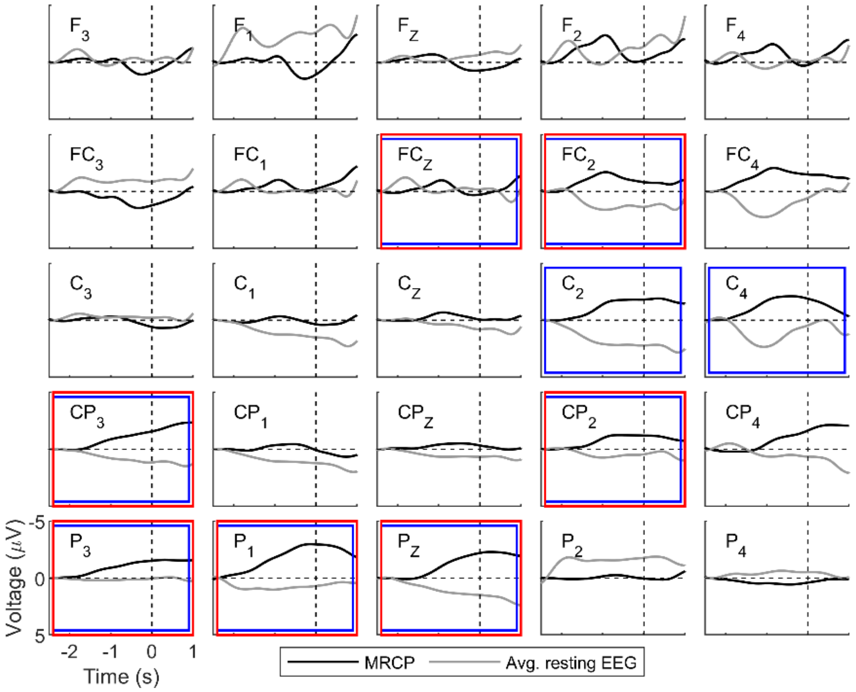

Fig. S4. Grand-averaged MRCPs measured during movement and rest trials for participant P2. Blue boxes indicate visually short-listed MRCP channels. Of these, channels retained by the automatic channel selection algorithm for training the BMI classifier, are encircled by a red box.

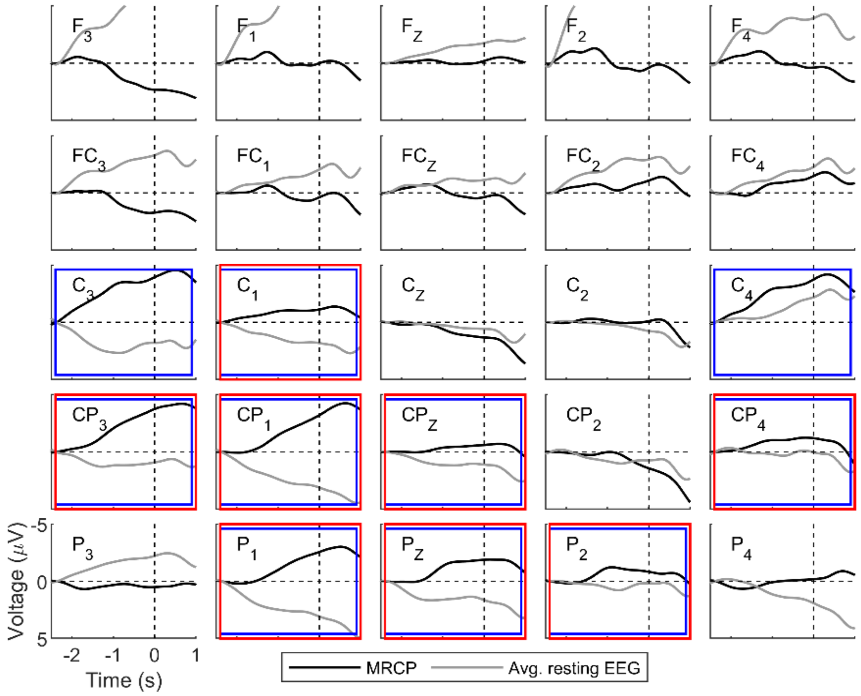

Fig. S5. Grand-averaged MRCPs measured during movement and rest trials for participant P3. Blue boxes indicate visually short-listed MRCP channels. Of these, channels retained by the automatic channel selection algorithm for training the BMI classifier, are encircled by a red box.

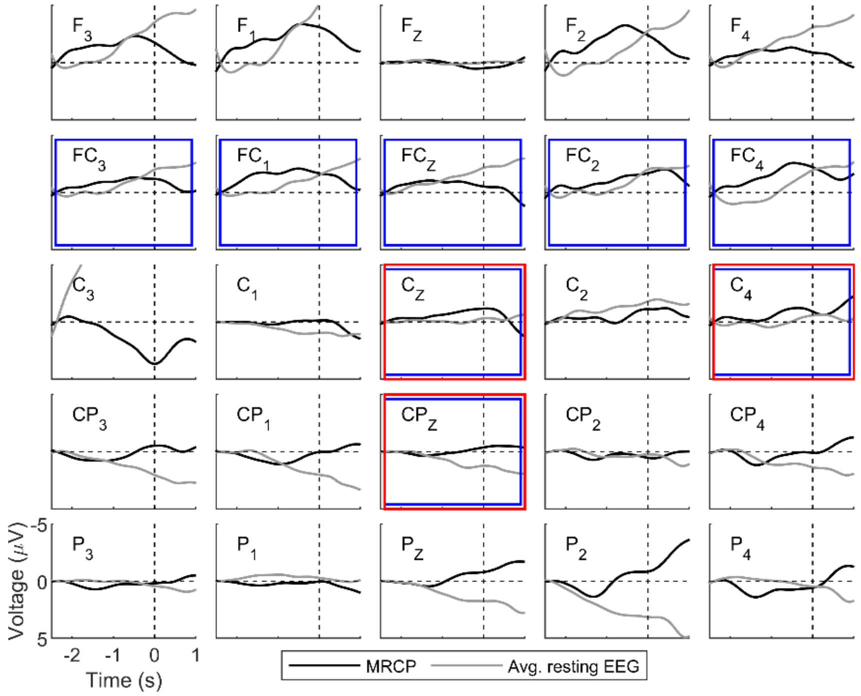

Fig. S6. Grand-averaged MRCPs measured during movement and rest trials for participant P4. Blue boxes indicate visually short-listed MRCP channels. Of these, channels retained by the automatic channel selection algorithm for training the BMI classifier, are encircled by a red box.

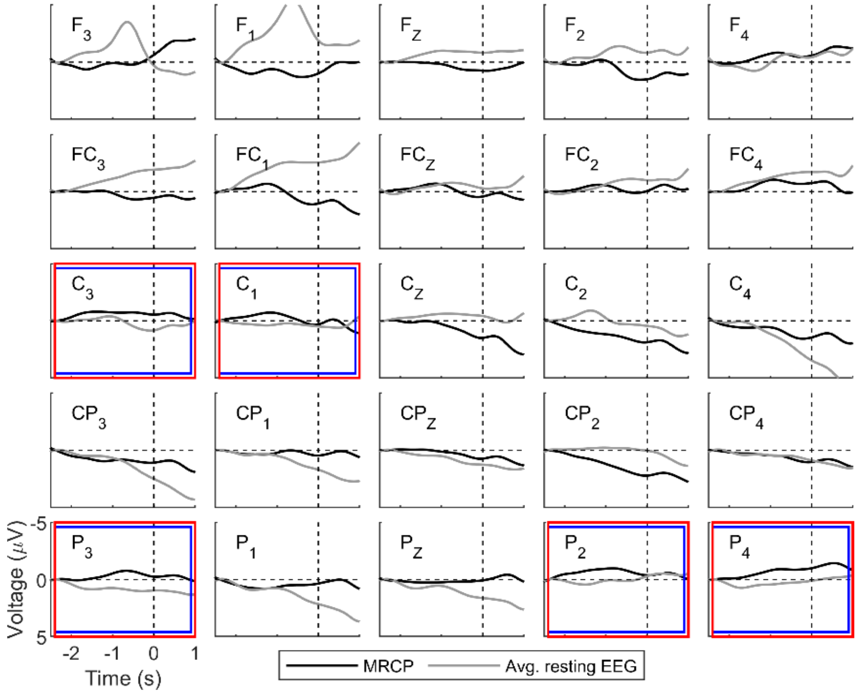

Fig. S7. Grand-averaged MRCPs measured during movement and rest trials for participant P5. Blue boxes indicate visually short-listed MRCP channels. Of these, channels retained by the automatic channel selection algorithm for training the BMI classifier, are encircled by a red box.

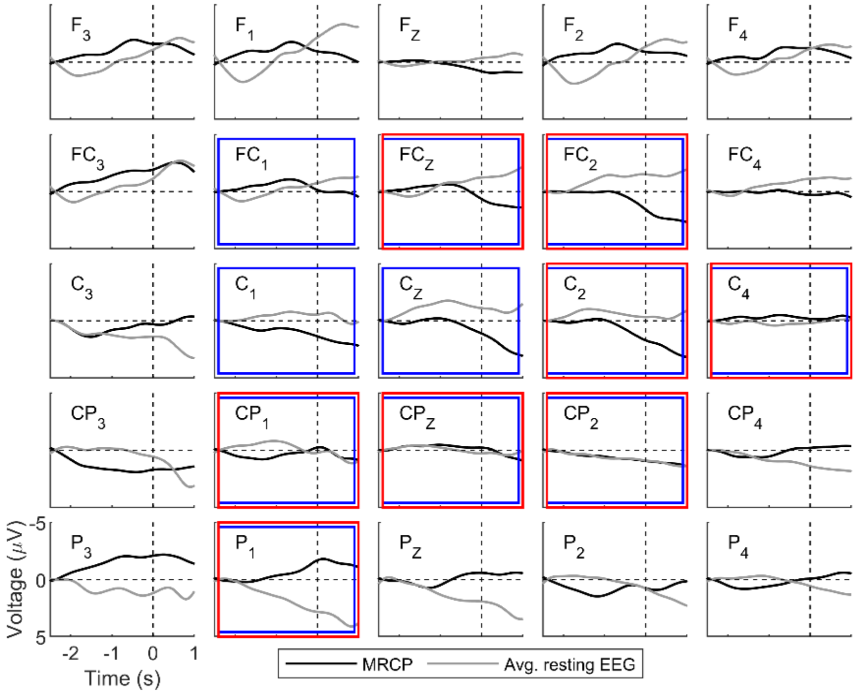

Fig. S8. Grand-averaged MRCPs measured during movement and rest trials for participant P6. Blue boxes indicate visually short-listed MRCP channels. Of these, channels retained by the automatic channel selection algorithm for training the BMI classifier, are encircled by a red box.

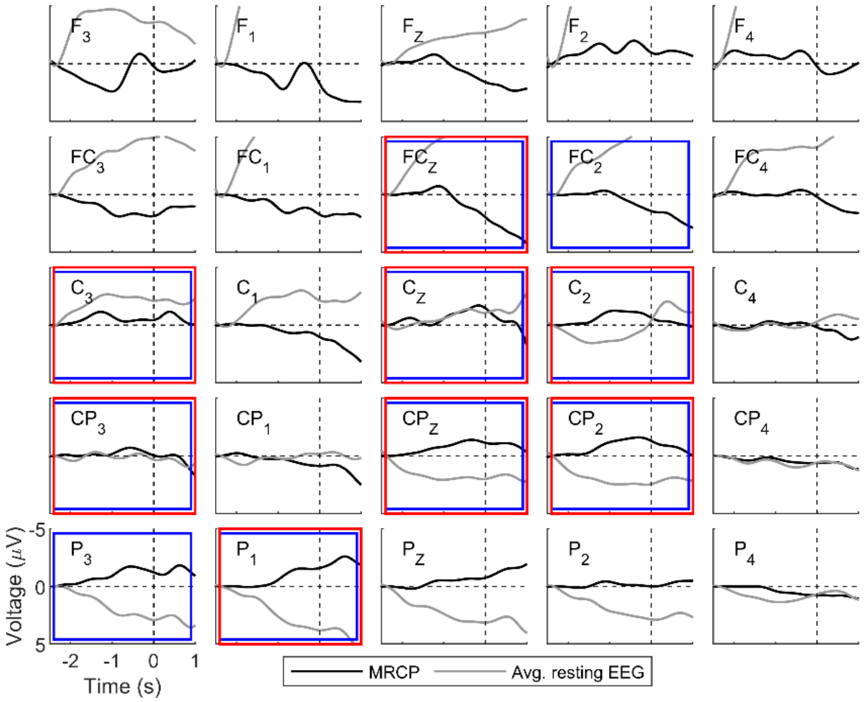

Fig. S9. Grand-averaged MRCPs measured during movement and rest trials for participant P7. Blue boxes indicate visually short-listed MRCP channels. Of these, channels retained by the automatic channel selection algorithm for training the BMI classifier, are encircled by a red box.

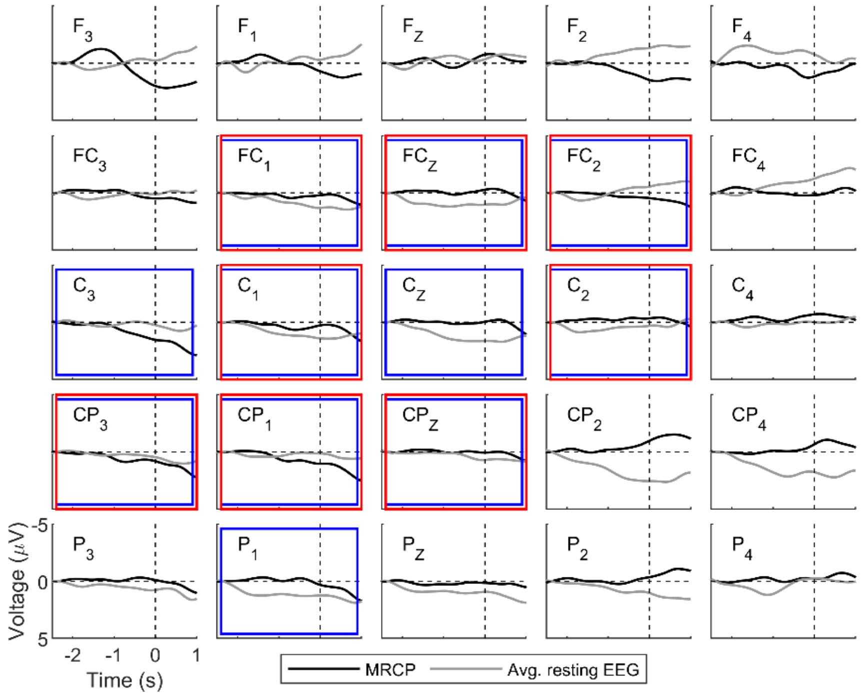

Fig. S10. Grand-averaged MRCPs measured during movement and rest trials for participant P8. Blue boxes indicate visually short-listed MRCP channels. Of these, channels retained by the automatic channel selection algorithm for training the BMI classifier, are encircled by a red box.

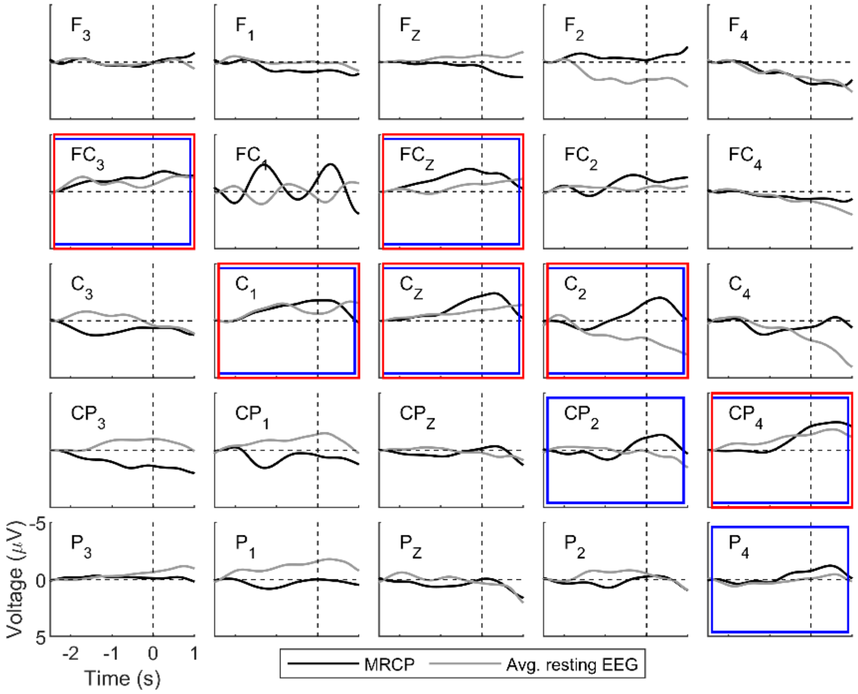

Fig. S11. Grand-averaged MRCPs measured during movement and rest trials for participant P9. Blue boxes indicate visually short-listed MRCP channels. Of these, channels retained by the automatic channel selection algorithm for training the BMI classifier, are encircled by a red box.

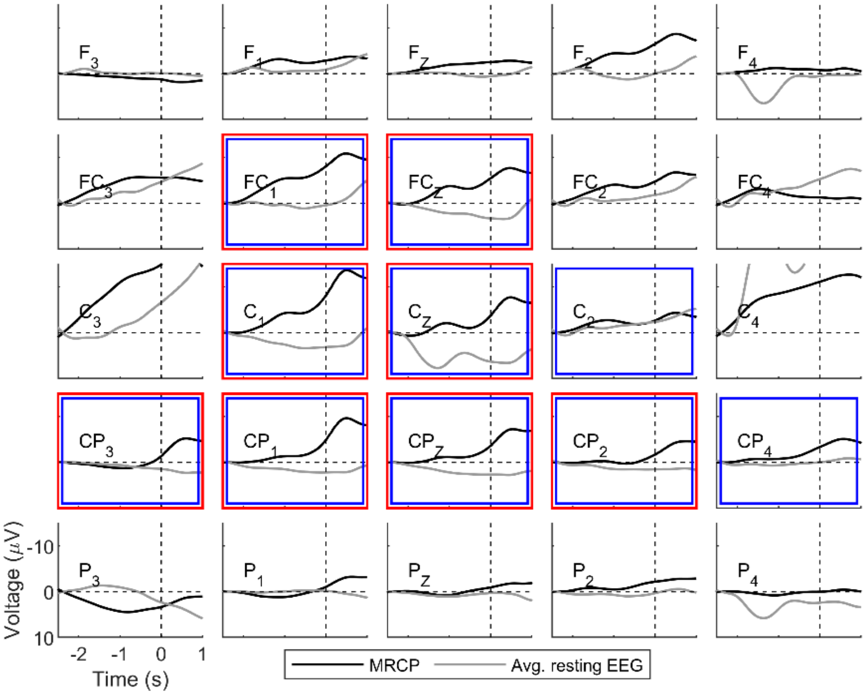

Fig. S12. Grand-averaged MRCPs measured during movement and rest trials for participant P10. Blue boxes indicate visually short-listed MRCP channels. Of these, channels retained by the automatic channel selection algorithm for training the BMI classifier, are encircled by a red box.
